# Comprehensive Review of the Literature on Existing Punctal Plugs for the Management of Dry Eye Disease

**DOI:** 10.1155/2016/9312340

**Published:** 2016-03-07

**Authors:** Naz Jehangir, Greg Bever, S. M. Jafar Mahmood, Majid Moshirfar

**Affiliations:** ^1^Francis I. Proctor Foundation, University of California San Francisco, San Francisco, CA 94143, USA; ^2^Department of Ophthalmology, Francis I. Proctor Foundation, University of California San Francisco, San Francisco, CA 94143, USA; ^3^Charles E. Schmidt College of Medicine, Florida Atlantic University, Boca Raton, FL 33431, USA; ^4^Department of Ophthalmology and Visual Sciences, Moran Eye Center, University of Utah, Salt Lake City, UT 84132, USA

## Abstract

Numerous designs of punctal and canalicular plugs are available on the market. This variety presents challenges to ophthalmologists when choosing punctal plugs for the management of various ocular conditions. The aim of this literature review is to provide a classification system for lacrimal occlusive devices based on their location and duration of action as well as to identify different characteristics of each one of them. We want to give a comprehensive overview on punctal and canalicular plugs including their manufacturing companies, indications, and complications that have been reported in various articles. PubMed and Google Scholar were used to identify articles written in English as well as few articles written in Japanese, Chinese, Slovak, and Spanish that had abstracts in English. Nine different companies that manufacture punctal and canalicular plugs were identified and their plugs were included in this review. Punctal and canalicular plugs are used in the management of various ocular conditions including dry eye disease and punctal stenosis as well as in ocular drug delivery. Although they are a relatively safe option, associated complications have been reported in the literature such as infection, allergic reaction, extrusion, and migration.

## 1. Introduction

Dry eye is a condition commonly seen by eye care practitioners; as many as 25% of patients seen in clinic have symptoms of dry eye [1]. The International Dry Eye Workshop (DEWS) defines dry eye as a multifactorial disease of tears and ocular surface with symptoms of visual disturbance, discomfort, and tear film instability with associated ocular inflammation and increased tear film osmolarity [2]. Data from Women's Health Studies (WHS) and Physicians' Health Studies (PHS) estimates 3.2 million women and 1.6 million men aged 50 years or older in the United States suffering from moderate to severe dry eye [3–5]. It is estimated that 8.5 million Americans spend more than 300 million dollars on artificial tear preparations and other related over-the-counter medications for dry eye disease [6]. The DEWS classified dry eye disease into four levels depending on severity of the disease and treatment options were recommended accordingly [7]. Topical lubricants, topical cyclosporine (Restasis), tetracyclines, and punctal plugs are a few of the available treatment options [8]. Plugs can be classified according to their location (punctal versus canalicular) and their duration of placement (temporary versus permanent). They are made of different materials that include collagen, silicone, hydrogel, polydioxanone, and acrylic. The ability to preserve tears makes them useful in certain cases of refractive surgery and contact lens intolerance [9]. Lacrimal occlusion with plugs prolongs the effects of lubricants and preserves natural tears. They are relatively contraindicated in patients with dry eyes and coexisting inflammation. Blocking the puncta exposes the ocular surface to tears having preexisting proinflammatory cytokines that worsen the ocular inflammation [10].

The use of punctal plugs is not limited to dry eye disease. Perforated punctal plugs have been successfully utilized in the treatment of punctal stenosis resulting in significant improvement in epiphora associated with the stenosis. Punctal plugs can be used for ocular drug delivery and can modulate the effect of other forms of topical treatment [11, 12]. This can be utilized in the treatment of glaucoma by increasing the drug retention time [9]. Both punctal and canalicular plugs have been associated with complications that have been reported in the literature. They can result in infections such as canaliculitis, biofilm formation, extrusion, migration, epiphora, and chronic irritation [9].

The purpose of this literature review is to give clinicians an update on different types of punctal and canalicular plugs, with recent advancements in designs and techniques. Choosing the best suitable punctal/canalicular plug for treatment of various ocular surface disorders (dry eye disease, punctal stenosis, epithelial erosions, and ocular drug delivery) may be difficult for clinicians as a large variety of punctal and canalicular plugs in different shapes, designs, and materials are available. It is important for the clinicians to be familiar with the complications that have been reported with different lacrimal plugs and to evaluate patients for any preexisting ocular or lid abnormalities. This paper provides a comprehensive overview of all the available punctal and canalicular plugs and can serve as a guide for clinicians to choose the most suitable lacrimal plug when treating the above-mentioned conditions.

## 2. Materials and Methods

PubMed and Google Scholar were searched for studies published up to October 2015. Eligibility criteria included studies evaluating indications, contraindications, adverse effects, shapes, designs, and characteristics of different punctal and canalicular plugs. Using Google, we searched for different manufacturers of punctal and canalicular plugs and used pictures (after getting permission from respective manufacturers) and characteristic features of the plugs to compile classification tables. We also evaluated different types of punctal and canalicular plugs microscopically to evaluate their characteristics. Keywords included punctal plugs, dry eyes, punctal stenosis, silicone plugs, perforated plugs, drug delivery, collagen plugs, SmartPlug, EagleFlex, Lacrimal Gland Occlusion, and intracanalicular plugs. A variety of articles related to punctal plugs were included in this review.

### 2.1. Classification of Punctal and Canalicular Plugs

**Figure 1:**
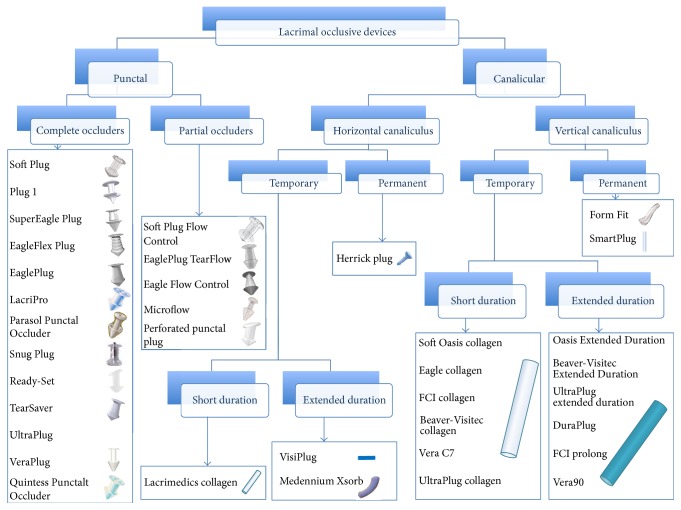
Classification of lacrimal occlusive devices based on shape, location, and duration of action.

**Figure 2:**
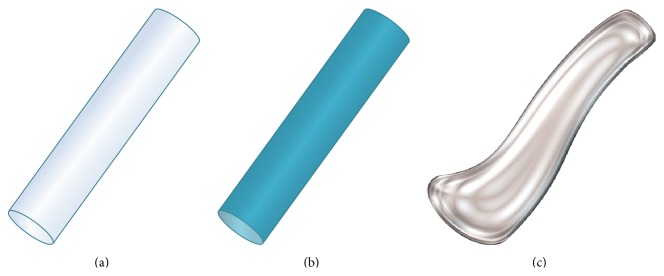
Vertical canalicular plugs. (a) Schematic of the temporary short duration plug made of collagen and effective for 4–14 days. (b) Representation of the temporary extended duration plug made of different materials (polydioxanone, glycolic acid and trimethylene carbonate, and E-Caprolactone-L-Lactide copolymer) and effective for about 2–6 months depending on the manufacturer. (c) Schematic of the hydrogel (Form Fit) plug that expands with hydration to mold into canaliculus and be permanently effective (*image C courtesy of* http://www.oasismedical.com).

Lacrimal occlusive devices can be classified into punctal and canalicular plugs (Figure 1). Freeman, in 1975, developed the dumbbell shaped punctal plug made of silicone and this concept of punctal plug is still in use [13]. Punctal plugs rest at the punctal opening making them easily visible and, hence, are removable without much difficulty. In contrast, canalicular plugs are not visible as they are placed inside the canaliculus (either the vertical or the horizontal canaliculus), making extrusion unlikely but increasing the risk of migration and difficulty in localizing their position without ultrasound [14]. Occlusion of the lacrimal drainage system with temporary or permanent plugs is a widely used nonpharmacological therapy for conserving tears. A wide variety of lacrimal plugs with specific indications are in use. Both horizontal and vertical canalicular plugs can be further classified into temporary and permanent. Temporary short duration canalicular plugs (Figure 2(a)) are used before attempting extended duration or permanent occlusion to assess risk of epiphora and the probability of symptomatic relief [15]. Temporary short duration ones plugs are usually made of animal collagen and last for 4–14 days. Temporary extended duration plugs (Figure 2(b)) are used following refractive surgery, for dry eye disease and for ocular retention of medications [16]. Temporary extended duration plugs can last from 2 to 6 months [17]. They are made of different materials such as glycolic acid with trimethylene carbonate, E-Caprolactone-L-Lactide copolymer (PCL), and polydioxanone (PDS).

### 2.2. Characteristics of Punctal Plugs

Different designs and shapes of plugs have been developed to increase their effectiveness and to minimize complications. Generally punctal plugs have a head on the top and are shaped like an umbrella. The head facilitates removal of the plug if necessary [26]. They usually have a slender neck and a cone-shaped thicker base. The majority of the punctal plugs are made of silicone, but teflon, hydroxyethylmethacrylate (HEMA), and polymethylmethacrylate (PMMA) have been tested [9]. We evaluated different punctal plugs under the microscope and classified them based on their shapes (Table 1). Punctal plugs have different shaft designs (e.g., tapered shafts and straight shafts) with pros and cons of different styles. The head portion can have reservoirs in some designs for increased trapping of tears. There are variations in the collarette such as a slanted collarette, which improves the fit. Some designs have tractional ribs for greater flexibility while some have collapsible noses that spring open once inside the puncta (Table 1). Perforated punctal plugs have a central lumen; they are used in treating punctal stenosis and partial occlusion by allowing some tear flow through the plug [27]. Punctal plug manufacturers, sizes, and characteristics are discussed in Table 2.

### 2.3. Characteristics of Canalicular Plugs

**Figure 3:**
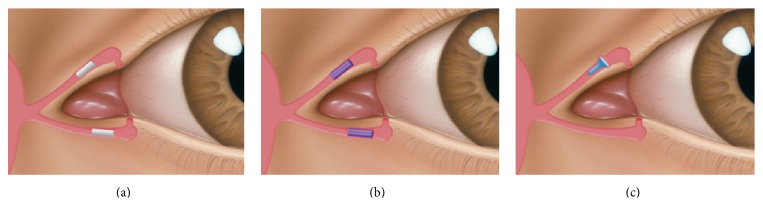
Horizontal canalicular plugs. (a) Collagen plug (Lacrimedics) that is meant to last for about two weeks. (b) Temporary extended duration plug (VisiPlug by Lacrimedics) made of polydioxanone does not swell with moisture and lasts about 6 months. (c) Permanent canalicular plug (Herrick plug by Lacrimedics) is shaped like a golf tee (*photos courtesy of* http://www.lacrimedics.com).

**Figure 4 fig4:**
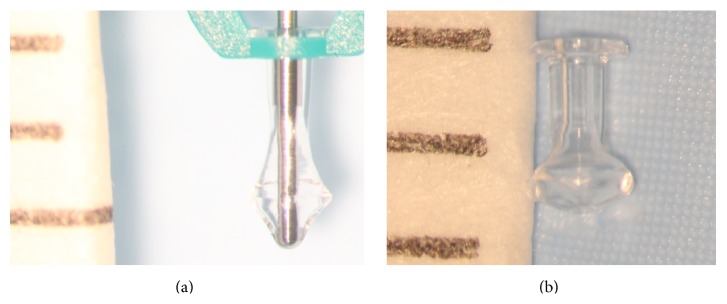
Snug Plug (FCI) is a punctal plug that is preloaded in a stretched position (a) compared to its natural shape (b) after release from inserter. The plug is on stretch for insertion with the goal of eliminating the step of punctal dilation prior to insertion. The widened bulb at the plug's base in the natural position acts to prevent the plug from falling out spontaneously. Each dash in scale represents 1 mm.

**Figure 5 fig5:**
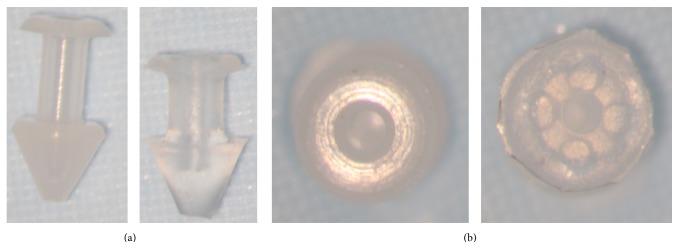
Punctal plug without reservoirs (VeraPlug) on (a) compared with punctal plug with reservoirs (Quintess Punctal Occluder) on (b). The Quintess Punctal Occluder was designed to have reservoir indentations in the collar to trap tears.

**Figure 6 fig6:**
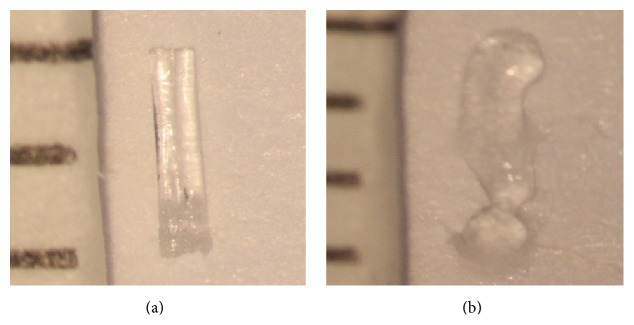
Hydrogel vertical canalicular plug (Form Fit by Oasis). Dry (a) compared to wet (b) demonstrates that the plug becomes more gelatinous and enlarges slightly following hydration (10 minutes after application of 0.2 mL of water). Each dash in scale represents 1 mm.

**Figure 7 fig7:**
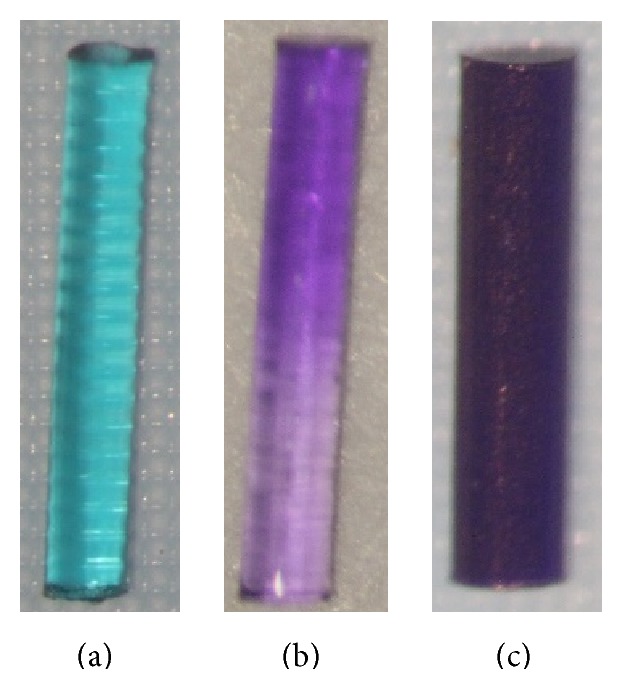
Comparison of extended duration plugs. (a) Soft Plug Extended Duration (Oasis)—extended duration temporary (3 months) plug made with a copolymer of glycolic acid and trimethylene carbonate. (b) Vera90 (Lacrivera)—extended duration temporary (3 months) plug made of E-Caprolactone-L-Lactide (PCL) copolymer. (c) VisiPlug (Lacrimedics)—extended duration temporary (6 months) plug made of polydioxanone (PDS).

**Figure 8 fig8:**
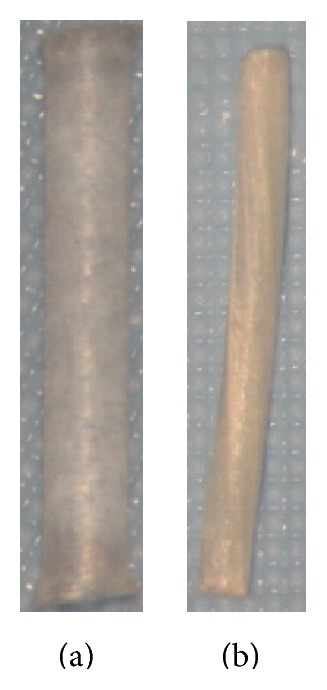
Comparison of temporary short duration plugs. (a) Lacrivera's Vera C7 made of collagen and effective for 7–10 days. (b) Oasis Soft Plug Collagen which is effective for 2–5 days.

Canalicular plugs for temporary use are usually rod-shaped and available in different sizes and colors depending on the punctal size. They are inserted into the canaliculus making it difficult to visualize them or monitor their position. To achieve complete occlusion of the lacrimal drainage system, the diameter of the plug is more important than its length [9]. Special designs for permanent use have been developed. The Form Fit plug (Figure 2(c)) is a vertical canalicular plug made of hydrogel that expands into a soft gelatinous material after contact with the tear film, filling and conforming to the shape of the vertical canaliculus [17]. Thermosensitive acrylic canalicular plugs (Medennium SmartPlug) have been in use since 2002. They become shorter and thicker at body temperature. The plug has a diameter of 0.4 mm and length of 9 mm before insertion that change after insertion to a diameter of 1 mm and a length of 2 mm [28]. Newer materials are thought to reduce bacterial adhesion and chances of infections [9]. Horizontal canalicular plugs can be temporary or permanent (Figure 3 [Fig fig4] [Fig fig5] [Fig fig6] [Fig fig7] [Fig fig8]). Herrick canalicular plugs, made of silicone, are placed in the horizontal canaliculus and shaped like a golf tee. They do not require punctal dilation prior to insertion. Removal of these horizontal canalicular plugs may prove challenging, in some cases, due to their intracanalicular location [29]. The Herrick plug is partially radiopaque and dyed blue in color to make localization possible with transillumination [9]. Horizontal and vertical canalicular plugs are discussed in Tables 3 and 4.

### 2.4. Indications of Punctal and Canalicular Plugs

*(i) Dry Eye Disease*. Occlusion of the lacrimal drainage system with plugs is considered an option in patients with moderate dry eye syndrome. An article by the American Academy of Ophthalmology reviewed literature to assess efficacy and safety of punctal and canalicular plugs for treatment of dry eye disease. The use of lacrimal plugs improved the symptoms, enhanced the ocular surface health, and decreased the use of lubricants in dry eye disease [30]. Recently a survey was sent to researchers and expert ophthalmologists in order to identify the common treatments used for managing dry eye disease [31]. It was observed that topical therapies are most commonly prescribed including steroids, cyclosporine A, and autologous serum. Among the nontopical therapies, respondents commonly use punctal plugs, tetracycline, flaxseed supplements, and essential fatty acid supplements. In another study, 86% patients were free of symptoms of dry eye at 6-month follow-up and 76% of patients had stopped using lubricants after punctal occlusion with silicone punctal plugs [32]. Punctal occlusion is effective in treating many conditions that cause dry eyes such as Stevens-Johnson syndrome, keratoconjunctivitis sicca, contact lens wear, and superior limbic keratoconjunctivitis [33]. Another study evaluated canalicular occlusion with collagen and silicone plugs (Herrick plugs) in patients with dry eye related conjunctivitis. It was a prospective, randomized trial and at the 8-week visit, there was a marked reduction in total dry eye (94.2%) and conjunctival symptom scores (93%) which was in sharp contrast to the sham group that experienced no change from the baseline [34]. Silicone punctal plugs have been associated with a significant decrease in tear film osmolality and a 75% decrease in rose bengal staining in 17 patients with dry eye [35]. Silicone punctal plugs used in keratoconjunctivitis sicca patients showed an improvement in goblet cell density, tear film stability, and ocular staining scores [36]. In another study, both collagen and silicone plugs resulted in an increase in aqueous tear volume and improved Schirmer I results, tear breakup time, and rose bengal staining [37]. Some studies have evaluated the SmartPlug in dry eye disease with a significant improvement in subjective symptoms and a decreased need for lubricants [38–40]. Kojima et al. reported no complications at 3-month follow-up after insertion of SmartPlug [39]. Although Schirmer test values were not significantly different before and after SmartPlug insertion, there was an improvement in rose bengal staining and a decrease in tear clearance rate. There is a possibility that these plugs do not fully occlude the canalicular lumen leading to an unchanged Schirmer test after plug insertion [39].

*(ii) Refractive Surgery*. Transient dry eye has been reported after laser surgery with a 59% incidence reported in a study 1 month after laser in situ keratomileusis (LASIK). Lacrimal plugs have a role in postrefractive surgery dry eyes and have been used preoperatively to prevent dry eye [41]. There are some controversies associated with the use of punctal and canalicular plugs in these scenarios. Occlusion can decrease the production of tears and reduce their clearance, which acts to worsen the dryness by increasing proinflammatory cytokines [42]. Yung et al. evaluated efficacy of punctal plugs in patients with post-LASIK dry eye [43]. The EaglePlug (EagleVision), a permanent silicone plug, was inserted a month after refractive surgery. Corneal sensitivity, Schirmer testing, and tear breakup time all improved in the treated group compared to the nontreatment group [43]. Albietz et al. reported that the use of lubricants and other options such as punctal plugs before refractive surgery increased the postprocedure goblet cell density [44]. A prospective randomized clinical trial evaluated punctal occlusion with punctal plugs after LASIK treatment for prevention of dry eye in 78 eyes of 39 patients [33]. Both eyes of the subjects underwent LASIK and lower punctal occlusion of one eye was performed while the other eye served as a control. At all follow-up visits the ocular surface index score was better and statistically significant for eyes with punctal plugs compared to the control eyes. At the 6-month final follow-up, although there was no statistically significant difference between the two eyes, the Schirmer I test, tear breakup time, and punctate epithelial keratitis scores were higher in the punctal plug occluded eyes than the control eyes. Kojima et al. evaluated preoperative insertion of punctal plugs to see its effects on postoperative vision and wound healing after laser epithelial keratomileusis [45]. Plugs were inserted both into the superior and inferior puncta. Significant improvement in the mean fluorescein score and mean uncorrected distance visual acuity was seen in the plug group compared to the nonplug group. Postoperative haze was less severe in the plug group. In another study, patients with low refractive errors (after refractive surgery) noted improvement in their visual acuity after silicone punctal plug placement [46]. Eighty-six percent of patients (7 eyes) gained at least one line of Snellen uncorrected visual acuity after punctal plug placement and decreased the desire to pursue further refractive surgery in 92% of the study group subjects. Collagen canalicular plugs can last from 3 days to 2 weeks and can improve symptoms of dry eye after laser refractive surgery by reducing flow through the canaliculus by 60–80% [47]. A literature review on prevention and treatment of LASIK-associated dry eye recommends treatment with artificial tears, punctal occlusion, topical cyclosporine A, and nutritional supplements prior to LASIK. It decreases the incidence of troublesome symptoms following laser surgery [48]. Huang at al. reported improvement in goblet cell density, corneal wound healing, and visual acuity in patients with temporary punctal occlusion after laser refractive surgery [49]. 

*(iii) Contact Lens Wearers*. Contact lens wearers with symptoms of dry eyes can benefit from punctal and canalicular plugs. Increased tear retention improves the symptoms of dry eye. Li et al. used ultrahigh resolution optical coherence tomography to see the effect of punctal occlusion on tear menisci in contact lens wearers with and without symptoms of dry eyes. Tear menisci increased transiently in both symptomatic and asymptomatic lens wearers and increased for a longer duration in symptomatic wearers [50]. A randomized controlled clinical trial evaluated the effect of punctal occlusion in dry eye contact lens wearers using a self-assessment questionnaire and evaluation of pre- and postlens tear film thickness. Extended wear intracanalicular plugs were used. Both the plug group and the sham group had significant improvement in their symptom scores. The effect of punctal occlusion did not differ between the two groups in terms of questionnaire score and treatment benefit assessment. It may indicate that punctal occlusion has no beneficial effect or the treatment effect was not detected due to a small sample size, nonparametric testing, or spontaneous plug extrusion [51]. Virtanen and colleagues observed a short lasting subjective and objective improvement in signs and symptoms after placement of horizontal canalicular plugs in contact lens wearers with both tear film deficiency and lens intolerance [52]. Intracanalicular plugs cannot be visualized directly making it difficult to exclude the possibility of migration or extrusion during the follow-up period. Improvement of symptoms and signs of dry eye disease was seen with insertion of silicone punctal plugs in a contact lens wearer with Sjogren's syndrome [53]. Silicone punctal plugs were inserted monocularly in lower puncta of 25 contact lens wearers with symptoms of dry eye [54]. Eighteen of the twenty-five patients reported a 34.6% increase in comfortable contact lens wear time at the 3-week follow-up. 

*(iv) Topical Medication Retention*. Sustained delivery of ocular medications in patients with glaucoma and dry eye disease is needed and punctal plugs can reduce the dose of the drugs by attaining effective drug concentration while minimizing the risk of side effects. Punctal plugs used for drug delivery are made from various polymers and composed of an optional cap containing pores, optional outer shell that is impermeable to the drug and tears, cylindrical body containing the drug compound, and an optional unit for retaining the plug over a long period of time. The cap can have one or more pores for the release of drugs and can extend throughout the body. The head portion rests on the exterior of the punctum and the bottom end is tapered or narrower for easy insertion [55]. Recently canalicular plugs made from thermosensitive, hydrophobic, acrylic material (SmartPlug) have been used for ocular drug delivery with better retention [56]. The latanoprost punctal plug delivery system has been recently used for treatment of primary open-angle glaucoma and ocular hypertension. It has completed a phase II clinical trial and has shown promising results [57]. An olopatadine punctal plug drug delivery system has been used in patients with allergic conjunctivitis but has not shown significant efficacy compared to placebo delivery system [58]. There are reports on cyclosporine and moxifloxacin releasing punctal plug models being developed and used for delivery of these drugs [59, 60]. This approach can improve the quality of life for many patients. 

*(v) Acquired Punctal Stenosis*. Konuk and colleagues evaluated perforated punctal plugs coated in PVP (polyvinylpyrrolidone) to treat complete and partial punctal stenosis in 44 eyes. The plugs were removed after 2 months with a mean follow-up period of 19 months. Success was achieved in 84.1% of eyes with relief of epiphora although a few cases had recurrence and mild horizontal lid laxity [27]. Chang et al. had similar results with a follow-up of more than 6 months [61]. Epiphora resolved in 85% patients. The patients with failure were all older than the success group and had associated chronic blepharitis. Wound healing occurring around the perforated punctal plug prevents restenosis. More prospective studies with larger sample sizes and longer follow-ups are needed to assess the effectiveness of perforated punctal plugs in treating partial and complete punctal occlusion. Punctal stenosis has also been treated with one-snip canaliculotomy and insertion of temporary punctal plugs to prevent restenosis [62]. 

*(vi) Superior Limbic Keratoconjunctivitis*. It has been observed that localized tear deficiency can cause friction between the upper lid and superior limbus resulting in symptoms of superior limbic keratoconjunctivitis (SLK). Upper punctal occlusion was used for management of refractory SLK and excellent results were obtained in all 22 eyes [63]. In one case report, administration of hydroxypropyl cellulose inserts improved symptoms of dry eye while SLK persisted in a patient with both Sjogren's syndrome and SLK. After several years of contact lens use the patient's symptoms reappeared and silicone punctal plugs were inserted, which improved both their dry eye disease and their superior limbic keratoconjunctivitis [53]. In another study SLK was an indication for placement of punctal silicone plugs in 11 eyes or 5.4% of the study group [64]. 

*(vii) Postkeratoplasty Astigmatism*. Collagen plugs have been implanted in radial keratotomy incisions to treat astigmatism after penetrating keratoplasty and eight of the eleven plugs were present several years later without any complications [65]. Espaillat et al. evaluated EagleVision collagen implants and treated high residual astigmatism after penetrating keratoplasty in 8 patients. Collagen plugs can be implanted as spacers between the relaxing incisions creating corneal flattening along the steep meridian. Although collagen implants usually do not last for more than a few days, Espaillat et al. observed that the implants can last up to 6 months in these grafts [66]. Collagen implants have been inserted in two live animal models with astigmatic keratotomy incisions and have been found to be safe and can enhance the effect of the incisions [67]. 

*(viii) Others*. Recurrent corneal erosions, epitheliopathy after penetrating keratoplasty, and persistent epithelial defects can also be managed with punctal and intracanalicular plugs. Tai et al. in a retrospective study observed that dry eye was the most common indication for silicone punctal plug insertion followed by epitheliopathy after penetrating keratoplasty (15.8%) [64]. Intraepithelial erosions during LASIK can be managed with punctal plugs, autologous serum drops, topical antibiotics, and bandage contact lenses. A female patient with a history of kidney disease developed recurrent epithelial erosions after LASIK and was managed with topical medications, soft bandage contact lens, and insertion of punctal plugs [68].

The indications of punctal and canalicular plugs are summarized in as follows.

*Indications of Punctal and Canalicular Plugs*

- Dry eye disease.
- Contact lens wearers.
- Punctal stenosis.
- Refractive surgery.
- Post keratoplasty.
- Topical medication delivery.
- Superior limbic keratoconjuctivitis.
- Recurrent corneal erosions.

### 2.5. Complications of Punctal and Intracanalicular Plugs

#### 2.5.1. Punctal Plugs

*(i) Extrusion, Granulation, Migration, and Enlargement of Punctal Size*. Extrusion has been commonly reported with silicone punctal plugs occurring at a rate of 25–50% reported over the course of a month to 2 years after placement of these devices [32, 64, 69]. Sonomura et al. investigated complications with the SuperEagle Plug (EagleVision). The study involved 148 puncta of 64 eyes. The extrusion rate was 57.4% in the follow-up period with no change in the size of the puncta or migration. Granulation was seen in 34.5% of patients [70]. A similar study done in Japan compared the EaglePlug, PunctalPlug, EagleFlex, and SuperFlex plugs to evaluate migration, extrusion, and enlargement of punctal size after extrusion in 291 eyes. They found that the time to extrusion was longer for SuperFlex plug than for others. Granulation tissue formed in 1.7% of the SuperFlex cases. In all the cases, a significant enlargement in the size of punctum was seen after extrusion [71]. Complete plug extrusion has the risk of enlarging the puncta, making reextrusion likely. A study on FCI punctal plugs with a slanted collarette was conducted with an observation period of 8 years. Retention rate was 84.2% after three months and decreased to 55.8% after a median of 2 years. Canalicular stenosis was seen after extrusion in 34.2% cases after 2 years. FCI plugs are harder than EaglePlugs (which are easier to insert and easy to remove) making retention better. It is also thought that the shape of FCI plug with the collarette with better fit lessens the foreign body sensation, minimizing the chances of extrusion [72]. Kaido et al. compared FCI silicone plugs with SuperFlex plugs (EagleVision) in a prospective interventional study [73]. The purpose was to investigate the retention rate and complications in relationship to the punctal size. Retention rate was 70.4% in the FCI plug F group compared to 30.1% with the SuperFlex at the 6-month follow-up. Spontaneous plug loss was attributed to a larger punctal size in patients with FCI plugs while old age with lid laxity was thought to be a contributory factor in patients with the SuperFlex. Punctal plug F is meant for insertion into puncta less than 0.8 mm in size. The high incidence of punctal plug extrusion has led to evolvement of new techniques to minimize the chances of this complication. Obata et al. described a technique to prevent reextrusion of punctal plugs. FlexPlugs of the same size as lost were sutured with 10-0 nylon in 10 puncta and 80% plugs were retained at 6 months [74]. To eliminate chances of plug migration, Kaido et al. used a plug size one diameter bigger than the measured punctal size. They inserted SuperFlex plugs and Soft plugs. No migration was seen at the 3-month follow-up period as compared to 13.8% with the standard technique [75]. In situations with severe dry eye and recurrent punctal plug extrusion, thermal cauterization is an effective treatment option with a very low recanalization rate [76]. Tai et al. reported a 49.4% retention rate of silicone punctal plugs with a mean survival time of 85.1 ± 7.3 weeks [64]. Most of the implants were lost within four weeks. Balaram et al. reported a 53% retention rate of punctal plugs after 6 months with a greater risk of extrusion in plugs placed in the upper versus lower puncta [32]. 

*(ii) Pyogenic Granuloma*. Pyogenic granuloma have been reported with both punctal and canalicular plugs. There is a case report of bilateral pyogenic granuloma with partial extrusion of perforated plugs in a patient 2 months after placement of the plug [77]. Musadiq et al. reported 2 cases of pyogenic granuloma occurring 3 months after insertion of Soft plugs [78]. Kim et al. in a retrospective observational case series with 903 silicone plugs (Parasol Punctal Occluders) observed pyogenic granuloma leading to extrusion of plugs in 4.2% of all the plugs placed. They proposed that formation of pyogenic granuloma could be due to irregular surfaces of silicone punctal plugs or the nose of plugs damaging the canalicular mucosa [79]. Pyogenic granuloma can develop anywhere in the body in response to injury or chronic irritation and silicone punctal plugs can cause this type of injury. An ampullary pyogenic granuloma overlying the superior punctum was reported in a female patient 14 months after placement of a silicone punctal plug. Both the plug and the granuloma were removed and a new silicone plug was inserted without complication [80]. 

*(iii) Punctal and Canalicular Stenosis*. Punctal plugs are used for reversible punctal occlusion and can cause punctal scarring and canalicular stenosis after extrusion or spontaneous loss. SuperEagle has been associated with canalicular stenosis in 34.2% of cases at 2 years [70]. Boldin et al. evaluated 17 eyes that developed punctal and canalicular stenosis after the loss of FCI punctal plugs and followed them up for a year [81]. The exact cause of stenosis was not known. It was thought to be attributed to the slanted collarette shape of the FCI plug damaging the punctal mucosa. This plug's unique shape necessitates rotation for a best fit. Other reasons postulated include collection of debris around the plug leading to chronic inflammation and scarring. It is thought that extrusion might be secondary to stenosis rather than stenosis secondary to extrusion. To determine the exact cause, larger studies need to be conducted and compare different punctal plugs to find any association of shape and designs with stenosis. 

*(iv) Canaliculitis and Dacryocystitis*. A study has reported 2 cases of spontaneous migration of EagleVision tapered shaft punctal plugs into the canalicular system causing canaliculitis and dacryocystitis [82]. Although chances of migration of punctal silicone plugs are less than canalicular plugs, it can still occur. Newer smaller sized plugs are more prone to distal migration and can lead to infection. Eye rubbing and a dilated punctum can be additional contributory factors. Another case of canaliculitis 30 months after punctal occlusion was reported in Japan [83]. Two cases of* Aspergillus fumigatus* infection with SuperFlex (EagleVision) and FCI silicone plugs were reported [84]. The exact cause of fungal infection was not known, but the possibility of the plug insertion being related could not be excluded. 

*(v) Epiphora*. Permanent punctal plugs have been associated with epiphora [85]. Epiphora has been reported in 10% of patients with punctal plugs in a report by the American Academy of Ophthalmology [30]. Another study reported epiphora in 11 eyes (5.4%) after insertion of silicone punctal plugs [64]. Shi et al. reported epiphora in 4 eyes (6.15%) [86]. 

*(vi) Biofilm Formation*. Punctal silicone plugs due to their exposed position and their complex shape can be easily contaminated with microbes resulting in an infection. In more than 50% of cases,* Staphylococcus* has been isolated from the culture of these contaminated plugs. There is some evidence that acrylic plugs may portend a lower risk of infection than silicone plugs [9]. It has been observed that the hole of punctal plugs can be associated with bacterial biofilm. Sugita et al. evaluated Ready-Set FCI punctal plugs with scanning electron microscopy and cultured material extracted from plugs for presence of bacteria in 21 patients with severe dry eye disease. Positive cultures were seen in 44% of the sample material extracted from the plugs.* Staphylococcus epidermidis* was the commonest organism isolated (75%) followed by* Staphylococcus aureus* (25%) [87]. It is very important to carefully monitor these plugs for any accumulation of material or related signs in order to prevent future infections. 

*(vii) Discomfort*. Localized discomfort has been associated with punctal plugs and some studies have reported this complication. Horwath-Winter et al. described localized discomfort with FCI silicone plugs in 2% of patients 34 months after placement of these plugs [72]. Balaram et al. reported localized discomfort with the EagleVision tapered shaft plug and the Oasis Soft Plug that was judged immediately and 3 months after plug placement [32]. Sugita et al. inserted silicone punctal plugs in 65 eyes and the most frequent complication observed was foreign body sensation [87]. 

*(viii) Punctal Plug Surface Defects*. The Quintess silicone punctal occluder with reservoir indentations was found to have punctal plug surface defects in 3 patients with local irritation of conjunctiva and inferonasal cornea [88]. These findings were observed 9, 40, and 69 months after their placement for symptoms of dry eye. These plugs were removed and found to have defect in collarette on scanning electron microscopy with sharp edges on the periphery. The irregular surface was also observed in the unused plugs under higher magnification. 

#### 2.5.2. Intracanalicular Plugs

*(i) Allergic Reaction*. Collagen absorbable plugs are made of bovine collagen, which is generally well tolerated. However, approximately 3% of the population is allergic to bovine collagen. Some studies have reported a granulomatous foreign body reaction with bovine collagen [89]. Ahn et al. reported a case of canaliculitis and a papilloma-like mass, three years after insertion of the plug [15]. Although collagen plugs usually dissolve in a few days, the possibility of retention cannot be excluded which mediates longer follow-up. 

*(ii) Canaliculitis, Dacryocystitis, and Other Infections*. Intracanalicular plugs are placed in the horizontal or vertical canaliculus and are made of different materials. The SmartPlug is made of thermosensitive acrylic material and has many advantages including minimal chance of foreign body sensation, corneal erosion, or extrusion given its intracanalicular location. Its removal is usually easy to achieve with lacrimal irrigation. A SmartPlug study group reviewed 28 patient charts with SmartPlug insertion and complications treated by ophthalmic and plastic reconstructive surgeons [90]. Of these 28 patients, 64.3% developed complications including canaliculitis, dacryocystitis, and conjunctivitis. Patients were managed differently depending on the severity of complications. Intracanalicular position can increase the chance of infection making removal of the plug necessary. Hill et al. reported the prevalence of canaliculitis to be 4.73% per SmartPlug inserted. The average time to develop symptoms after insertion was 3 years. The patients were treated with canaliculotomy and plug removal [91]. Plug removal by irrigation failed in all cases; thus surgical intervention was necessary for every eye. The control group with punctal plugs had a lower complication rate of 2.1% at 2-year follow-up. Gerding et al. reported bilateral canaliculitis in a patient 2 years after placement of Herrick plugs. Surgical intervention and resection of cicatrized canaliculi were performed [92]. Lacrimal irrigation is considered an option for plug removal, but it is not always effective and can cause more inflammation resulting in scarring and worsening of the infection. Hill et al. suggested canaliculotomy for removal of these plugs, but this procedure has its own complications. M. Zhang and X. Zhang recently suggested a new method for removal of SmartPlugs. They used a lid clamp to flip the lid outward and if the size of the puncta was large enough no tools were needed for removal of the plug. If the size of the puncta was small, micro forceps were used for punctal dilation before application of the lid clamp, making removal easy [28]. Mazow et al. have reported canaliculitis occurring more frequently with intracanalicular plugs than the punctal ones [93]. Theoretically, canalicular position makes the removal of the plugs easier by irrigation, but this may not be the case as the plug can get lodged in the lower canalicular system and increase the chances of complications. Sixty-six (6.9%) out of nine hundred ninety-eight surgical cases developed complications (60 Herrick plugs, 6 SmartPlugs) requiring removal of the plugs. Five eyes developed canaliculitis and 29 eyes developed dacryocystitis and needed surgical treatment. Rabensteiner et al. compared SmartPlugs with silicone punctal plugs in the treatment of dry eye with a follow-up period of 3 months and found no significant difference between the two groups [26]. They reported that the SmartPlug does not fully occlude the canalicular lumen and, thus, allows tears to pass through. Chen and Lee reported significant improvement in dry eye symptoms in 91 eyes of 54 patients after SmartPlug insertion, but canaliculitis was reported in 6 eyes [38]. A survey was undertaken involving the American Society of Ophthalmic and Plastic and Reconstructive Surgery (ASOPRS) members' experiences with Herrick plugs. Among the 61% respondents that reported complications after plug placement, only 25% reported successful plug removal with lacrimal irrigation. Cases have been reported where a patient had multiple silicone intracanalicular plugs placed in the past and developed* Nocardia* canaliculitis, dacryocystitis, and subperiosteal abscess. A second patient developed dacryocystitis needing surgery [94]. Complications have also been associated with Form Fit plugs placed in the vertical canaliculus. Joganathan et al. reported 3 cases with complications of* Klebsiella* canaliculitis, canalicular abscess, and granulation tissue [95]. Ultrasound biomicroscopy can be used as an efficient diagnostic tool to visualize position of a retained intracanalicular plug [96]. 

*(iii) Pyogenic Granuloma*. There is a case report of pyogenic granuloma developing 2 years after insertion of the SmartPlug [97]. In a similar case report, a 65-year-old female patient developed a pyogenic granuloma in her left eye three years after insertion of bilateral SmartPlugs. Two weeks later a new granuloma appeared and both the plug and granuloma were removed [98]. A 47-year-old female patient developed ampullary pyogenic granuloma over the left superior punctum after insertion of silicone lacrimal plug. The plug had migrated to the common canaliculus and had to be removed surgically [99]. A retrospective study evaluated 66 eyes with complications after placement of Herrick plugs and SmartPlugs; pyogenic granulomas were observed in 11% of the eyes [93]. 

*(iv) Epiphora*. White et al. reported complications related to Herrick plugs in 41 patients who had symptomatic epiphora after plug insertion. Simple irrigation was not able to remove the plug and in most cases dacryocystorhinostomy was performed [100]. One theoretical advantage of Herrick plugs is easy removal by lacrimal irrigation; however this can be difficult leading to permanent obstruction of the lacrimal drainage system. Jones et al. observed that 10% of patients with Herrick plugs underwent an adverse event; epiphora was the most common followed by plug migration. Epiphora resolved with plug removal with saline flush in all but three patients [101]. Epiphora requiring plug removal was reported in 5.5% eyes after SmartPlug insertion [38]. Epiphora has been reported with canalicular plugs in another retrospective study [93]. Complications of punctal and intracanalicular plugs are summarized in Table 5.

*(v) Plug Extrusion and Distal Migration*. Due to the intracanalicular position of these plugs, there is a lower risk of extrusion compared to punctal plugs. Chen and Lee evaluated SmartPlugs in 91 eyes and reported spontaneous plug loss in 2 eyes [38]. Distal migration has been associated with canalicular plugs. Soparkar et al. reported distal migration of permanent lacrimal plugs in 12 patients causing symptoms that warranted removal [102]. Mazow et al. had reported lodged intracanalicular plugs causing lacrimal obstruction in 66 eyes [93].

### 2.6. Contraindications of Punctal and Intracanalicular Plugs

The use of these lacrimal occlusive devices is contraindicated in patients who are allergic to any of the materials as well as in patients with lacrimal outflow obstruction, ectropion, and active ocular infection [30]. Infectious conjunctivitis, in particular, is a contraindication to the use of punctal plugs [64]. Severe inflammatory changes of the ocular surface and the lids (such as blepharitis) should be treated prior to insertion of punctal plugs to reduce proinflammatory cytokines that can exacerbate inflammation [9].

## 3. Conclusion

A wide variety of punctal and canalicular plugs are available in the market. Their use is not only limited to nonpharmacological management of dry eyes but is gaining popularity in several other ophthalmic diseases. Newer designs are being made to decrease the risk of complications. Nevertheless there are limitations of these plugs and close monitoring is needed after placement. Future studies are needed comparing different types of plugs and following outcomes over longer timeframes. With new technology and ongoing research punctal plugs will continue to have an important role in the management of a myriad of eye conditions.

## Figures and Tables

**Table 1:** Shapes of punctal plugs. Several designs are made by different companies to increase the effectiveness of punctal plugs with different shapes of the shafts, collapsible noses, reservoirs, and traction ribs.

Design	Advantages	Name	Model
Tapered shaft	Designs extra force horizontally to keep plug in place.	EaglePlug	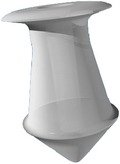
		TearSaver	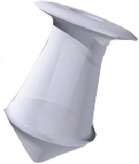
		SuperEagle Plug	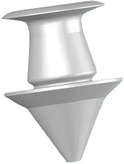

Collapsible nose	Collapsible hollow nose adheres the plug to the shape of ampulla and springs open once inside the puncta.	Parasol Punctal Occluder	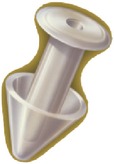

Reservoired head	Indentations trap tears and minimize foreign body sensation.	LacriPro	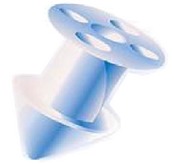
		Quintess Punctal Occluder	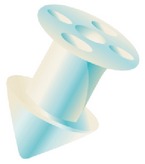

Ribbed shaft	Greater flexibility.	EagleFlex Plug	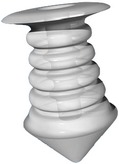
		SuperFlex	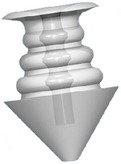

Perforated shaft	Slanted collarette perforated lumen.	Soft Plug Flow Control	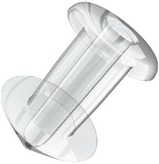
		Eagle Flow Control	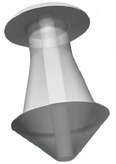
		EaglePlug TearFlow	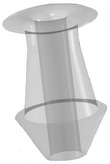
		Microflow	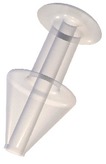
		Perforated punctal plug	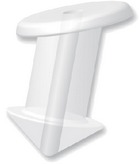

Slanted lip	Resists migration and prevents rubout. Conforms to the natural anatomy of eyelid.	Ready-Set	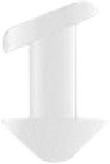

Dual lobe tip	Fits a range of punctal sizes.	Plug 1	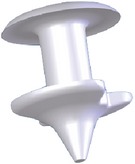

Stretched shaft	Returns to natural shape after insertion.	Snug Plug	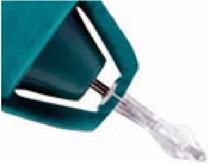

Straight shaft	Low profile dome and easy insertion.	VeraPlug	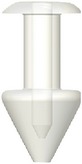
		Parasol Punctal Occluder	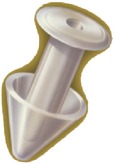
		Soft Plug	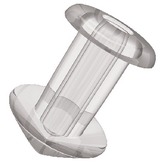
		UltraPlug	

**Table 2:** Punctal plugs [18–25] (types of punctal plugs, manufacturers, and characteristics discussed in detail).

Name	Size	Material	Characteristics	Manufacturer
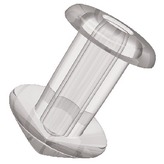 Soft Plug	5 sizes (0.4–0.8 mm) (micro, mini, petite, small, and medium)	Medical grade silicone	Pointed nose simplifies insertion; large anchor secures plug.	Oasis

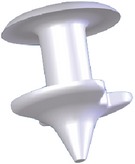 Plug 1	1 size (fits 0.5–0.8 mm puncta)	Silicone	Dual lobed design to fit a range of punctal sizes.	EagleVision

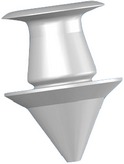 SuperEagle Plug	Small (0.4–0.6 mm) Medium (0.6–0.8 mm) Large (>0.8 mm) Values = puncta size	Low durometer silicone	Low durometer silicone with low profile rim for more comfort. Tapered shape and wide flexed nose design with a goal of good retention.	EagleVision

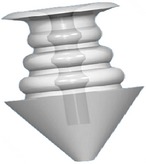 SuperFlex Plug	11 sizes (combinations with 0.3–1.3 mm diameter, 1.1–2.0 mm length)	Silicone	Better fit with good retention. Easy insertion.	EagleVision

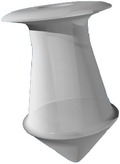 EaglePlug	5 sizes (0.4–0.8 mm)	Silicone	Tapered shaft exerts horizontal force to keep plug in place. Easy insertion and removal.	EagleVision

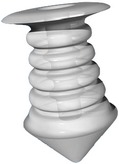 EagleFlex Plug	6 sizes (0.4–0.9 mm)	Silicone	Tapered shaft with traction ribs for better retention and flexibility. External ribs give 30% more surface area. Thin rim decreases corneal contact.	EagleVision

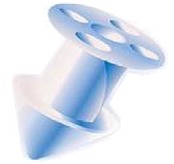 LacriPro Punctum	4 sizes (0.3, 0.5, 0.7, and 0.9 mm) (X-small, small, medium, and large)	Medical grade silicone	Reservoir indentations trap tears and decrease foreign body sensation. The number of indentations corresponds to the size of the plug.	Lacrimedics

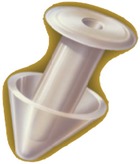 Parasol Punctal Occluder	X-small (0.25–0.35 mm) Small (0.35–0.65 mm) Medium (0.6–0.85 mm) Large (>0.9 mm) Values = puncta size	Silicone	Hollow nose. Umbrella-like head which squeezes as it is inserted and springs open once in place. No need for punctal dilation.	Beaver-Visitec

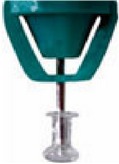 Snug Plug	1 size fits all	Silicone	Preloaded in a stretched position and returns to natural shape after insertion (Figure 4).	FCI

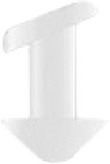 Ready-Set	7 sizes (0.4–1.0 mm) (slim mini, slim petite, micro, mini, small, medium, and large)	Silicone	Slanted lip in sizes 0.6 mm and larger makes it a better fit. Conforms to natural anatomy of the eyelid. Sizes 0.6 mm and larger also have slightly larger bulbs to resist migration and prevent rubout.	FCI

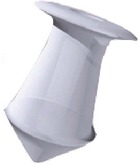 TearSaver	5 sizes (0.4–0.8 mm)	Silicone	Tapered shaft exerts extra horizontal force to keep plug in place.	FCI

UltraPlug	5 sizes (0.4–0.8 mm)	Silicone	Straight shaft. Low profile cap design minimizes foreign body sensation.	Surgical Specialties Corporation

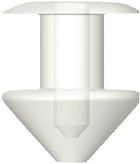 VeraPlug	Small (0.4–0.6 mm) Medium (0.6–0.7 mm) Large (0.7–0.8 mm) X-Large (0.8–1.0 mm) Values = puncta size	Silicone	Low profile dome for more comfort. Proprietary shaft design for easy insertion and good fit (Figure 5).	Lacrivera

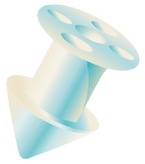 Quintess Punctal Occluder (AquaFlo)	4 sizes (0.3, 0.5, 0.7, 0.9 mm) (X-small, small, medium, and large)	Silicone	Reservoir indentations are designed to decrease foreign body sensation. Indentations correspond to plug size (Figure 5).	AlphaMed

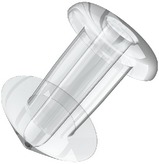 Soft Plug Flow Control	3 sizes (0.6–0.8 mm)	Soft silicone	Partial occluder. Opening through nose portion for partial occlusion. Low profile dome head. Softer, flexible silicone makes it more comfortable. Pointed nose secures it firmly. Patent lumen allows for use in punctal stenosis.	Oasis

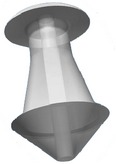 Eagle Flow Controller	4 sizes (0.5–0.8 mm)	Silicone	Tapered shaft. Partial occluder. Patent lumen allows for use in punctal stenosis. Tapered shaft increases vector force to keep the plug in position.	EagleVision

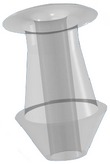 EaglePlug TearFlow	6 sizes: 0.5 mm plug, 0.2 mm lumen 0.6 mm plug, 0.3 mm lumen 0.7 mm plug, 0.3 mm lumen 0.8 mm plug, 0.3 mm lumen 0.9 mm plug, 0.4 mm lumen 1.0 mm plug, 0.5 mm lumen	Silicone	Partial occluder. Lumen size is 150% larger than Eagle Flow Control plug allowing for increased tear flow. Tapered shaft gives more controlled retention. Patent lumen allows for use in punctal stenosis.	EagleVision

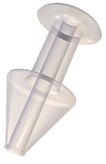 Microflow	Small (0.4–0.55 mm) Medium (0.55–0.7 mm) Large (0.7–0.85 mm) Values = punctal size	Silicone	Partial occluder. Patent lumen allows for use in punctal stenosis.	Beaver-Visitec

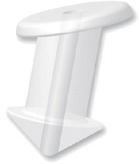 Perforated punctal plug	2 sizes (0.7, 0.9 mm) (mini, medium)	Silicone coated with PVP (polyvinylpyrrolidone)	Partial occluder. Slanted collarette. PVP (polyvinylpyrrolidone) is hydrophobic and allows tear to flow smoothly through the perforation. Used in treating punctal stenosis.	FCI

**Table 3:** Vertical canalicular plugs [18–25] (types of vertical canalicular plugs, manufacturers, and characteristics discussed in detail).

Name	Size	Material	Temporary/ permanent	Characteristics	Manufacturer
Form Fit	1 size (diameter 0.3 mm, length 3 mm)	Hydrogel	Permanent	Hydrates over 10 minutes after insertion and expands (Figure 6). With hydration it increases in size till it completely fills vertical canalicular cavity. It has low extrusion rate.	Oasis

SmartPlug	1 size (diameter 0.4 mm, length 6 mm)	Thermosensitive acrylic material	Permanent	Adjusts to shape and size of punctum. It shrinks to 1 mm after insertion to make it more comfortable by eliminating foreign body sensation. Less chance of extrusion. Can be flushed out with irrigation.	Medennium

Oasis Soft Plug Extended Duration	4 sizes (0.2–0.5 mm)	Glycolic acid and trimethylene carbonate	Temporary—extended duration	Effective up to 3 months (Figure 7(a)).	Oasis

DuraPlug	3 sizes (0.2–0.4 mm)	PCL (E-Caprolactone-L-Lactide copolymer)	Temporary—extended duration	Lasts 2–6 months.	EagleVision

Beaver-Visitec Extended Duration	4 sizes (0.2–0.5 mm)	Glycolic acid and trimethylene carbonate	Temporary—extended duration	Dyed with D&C Green. Lasts up to 3 months.	Beaver-Visitec

ProLong	3 sizes (0.3–0.5 mm)	Glycolic acid and trimethylene carbonate	Temporary—extended duration	Dyed with D&C Green number 6. Lasts up to 3 months.	FCI

Vera90	3 sizes (0.2–0.4 mm)	PCL (E-Caprolactone-L-Lactide copolymer)	Temporary—extended duration	Dyed violet with D&C Violet number 20 and is coated with calcium stearate (a noncollagenous and nonantigenic coating). Lasts up to 3 months (Figure 7(b)).	Lacrivera

UltraPlug Extended Wear	3 sizes (0.2–0.4 mm)	PCL (E-Caprolactone-L-Lactide copolymer)	Temporary—extended duration	Effective for 2–6-month duration.	Surgical Specialties Corporation

Oasis Soft Plug Collagen	3 sizes (0.2–0.4 mm) 2 mm length.	Collagen	Temporary—short duration	Lasts 2–5 days (see Figure 8(a)).	Oasis

Eagle collagen	3 sizes (0.2–0.4 mm)	Collagen	Temporary—short duration	Effective for 3–5 days and lasts for 7–10 days. Expands in punctum after insertion.	EagleVision

Beaver-Visitec collagen plug	3 sizes (0.2–0.4 mm)	Collagen	Temporary—short duration	Lasts 7–10 days.	Beaver-Visitec

FCI collagen plug	3 sizes (0.2–0.4 mm)	Collagen	Temporary—short duration	Lasts 5–7 days.	FCI

Vera C7	3 sizes (0.2–0.4 mm)	Collagen	Temporary—short duration	Effective for 7–10 days (Figure 8(b)).	Lacrivera

UltraPlug collagen	3 sizes (0.2–0.4 mm)	Collagen	Temporary—short duration	Effective for 10–14 days.	Surgical Specialties Corporation

**Table 4:** Horizontal canalicular plugs [18–25] (types of vertical canalicular plugs, manufacturers, and characteristics discussed in detail).

Name	Size	Material	Temporary/ permanent	Characteristics	Manufacturer
Herrick plug	3 sizes (0.3, 0.5, and 0.7 mm)	Medical grade silicone	Permanent	Shape of a golf tee and radiopaque. More comfortable initially but, because of the stagnant column of tear fluid between the plug and punctal opening, are theoretically more prone to infection. It has a collapsible bell design, which makes insertion easier.	Lacrimedics

VisiPlug	2 sizes (0.4–0.5 mm)	Polydioxanone (PDS)	Temporary—extended duration	Does not swell upon coming in contact with moisture (Figure 7(c)). Lasts up to 6 months.	Lacrimedics

Xsorb Plug	3 sizes (0.3–0.5 mm)	Glycolic acid and trimethylene carbonate	Temporary—extended duration	Dyed with D&C Green number 6. Lasts for about 3 months.	Medennium

Lacrimedics collagen	3 sizes (0.3–0.5 mm)	Collagen	Temporary—short duration	Lasts 4–7 days.	Lacrimedics

**Table 5:** Complications of punctal and intracanalicular plugs.

Type of plug	Complications
Punctal plugs	(i) Extrusion (most common)
	(ii) Granulation tissue
	(iii) Enlargement of punctal size
	(iv) Migration (less common than canalicular plugs)
	(v) Canalicular stenosis
	(vi) Foreign body sensation
	(vii) Pyogenic granuloma
	(viii) Canaliculitis
	(ix) Dacryocystitis
	(x) Fungal/bacterial infections
	(xi) Epiphora
	(xii) Corneal ulceration

Canalicular plugs	(i) Allergy
	(ii) Granulomatous foreign body reaction
	(iii) Canaliculitis and dacryocystitis (more common than punctal plugs)
	(iv) Difficult removal
	(v) Klebsiella canaliculitis
	(vi) Pyogenic granuloma
	(vii) Epiphora
	(viii) Migration
	(ix) Canalicular stenosis
